# A215 INJECTION SITE PAIN AND ADHERENCE IN PATIENTS SWITCHING FROM REFERENCE ADALIMUMAB TO AVT02 – INTERIM RESULTS OF THE EASE PAIN TRIAL.

**DOI:** 10.1093/jcag/gwae059.215

**Published:** 2025-02-10

**Authors:** D K Shahrokh, W Afif, A Steinhart, N Narula

**Affiliations:** Medical, JAMP Pharma Corporation, Boucherville, QC, Canada; McGill University Health Centre, Montreal, QC, Canada; University of Toronto Temerty Faculty of Medicine, Toronto, ON, Canada; McMaster Children’s Hospital, Hamilton, ON, Canada

## Abstract

**Background:**

AVT02, a biosimilar to reference product (RP) adalimumab, was first approved in Canada in 2022. AVT02 is formulated at high concentration and with citrate-free excipients. The EASE PAIN trial is a Phase IV study in Canada evaluating the impact of switching from RP, or an alternative adalimumab biosimilar, to AVT02.

**Aims:**

The primary objective was to examine the impact of switching on injection site pain across patients in Canada. Secondary objectives included describing the impact of switching on injection site reaction including burning sensation and soreness, treatment adherence, and patient-reported quality of life.

**Methods:**

The study enrolled patients with gastrointestinal conditions (Crohn’s disease [CD], ulcerative colitis [UC]), rheumatological conditions (rheumatoid arthritis [RA], ankylosing spondylitis [AS], psoriatic arthritis [PsA]), or dermatological conditions (hidradenitis suppurativa [HS], psoriasis [PsO]). Participants were eligible if their treating physician had decided to switch them from low-concentration RP or alternative adalimumab biosimilar to AVT02. The study assesses injection site pain measured via Visual Analog Scale (VAS), adherence, and quality of life (based on EQ-5D-5L) for participants up to Day 180 after switching. The study is currently ongoing (331 Patients enrolled in the Study; 287 included in the interim analysis)

**Results:**

The intention-to-treat (ITT) population comprised 287 participants. Following the first administration of AVT02, injection site VAS pain score decreased by an average of -18.8 ± 25.51 across the whole population. Supporting this, there was reduction in overall injection site reactions (49.1%), burning sensation (79.4%), and soreness (84.8%) between baseline (RP administration) and follow-up (AVT02 administration). Adherence rate was 95.5% overall. The ITT population maintained high quality of life score (EQ-5D-5L score of >81 on a scale of 1-100) after switching from RP to AVT02.

**Conclusions:**

The results of this study demonstrate that switching from RP or alternative biosimilar adalimumab to AVT02 leads to decreased injection site pain across all indications, and maintenance of high adherence rates and high quality of life among patients.

**Funding Agencies:**

None

